# 5-Bromo-2-(phenyl­amino)­benzoic acid

**DOI:** 10.1107/S2414314624001986

**Published:** 2024-03-06

**Authors:** Liping Kang, Sihui Long

**Affiliations:** a School of Chemical Engineering and Pharmacy, Wuhan Institute of Technology, Wuhan, Hubei 430205, People’s Republic of China; University of Aberdeen, United Kingdom

**Keywords:** synthon, hydrogen bond, acid-acid dimer, crystal structure

## Abstract

The title compound, C_13_H_10_BrNO_2_, was obtained by the reaction of 2,5-di­bromo­benzoic acid and aniline. The mol­ecule is twisted with a dihedral angle between the aromatic rings of 45.74 (11)° and an intr­amolecular N—H⋯O hydrogen bond is seen. In the crystal, pairwise O—H⋯O hydrogen bonds generate carb­oxy­lic acid inversion dimers.

## Abstract 2

The mol­ecules of the title compound pair up to form carb­oxy­lic acid–carb­oxy­lic acid homodimers in the crystal structure.

## Graphical abstract

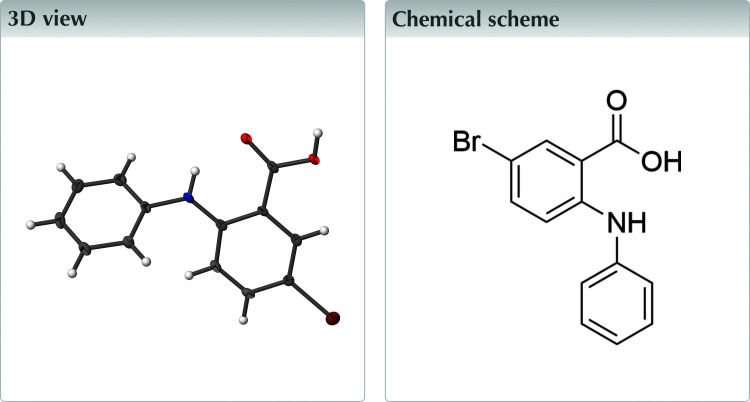

## Structure description

Non-steroidal anti-inflammatory drugs are among the most widely used drugs in the world (Enthoven *et al.*, 2017 ▸). These have anti-inflammatory, anti­pyretic and analgesic effects and can be sold as prescription drugs and over-the-counter drugs for the treatment of fever, acute or chronic pain and a variety of inflammatory diseases such as osteo­arthritis, rheumatoid arthritis, *etc* (Machado *et al.*, 2021 ▸).

**Figure 1:**
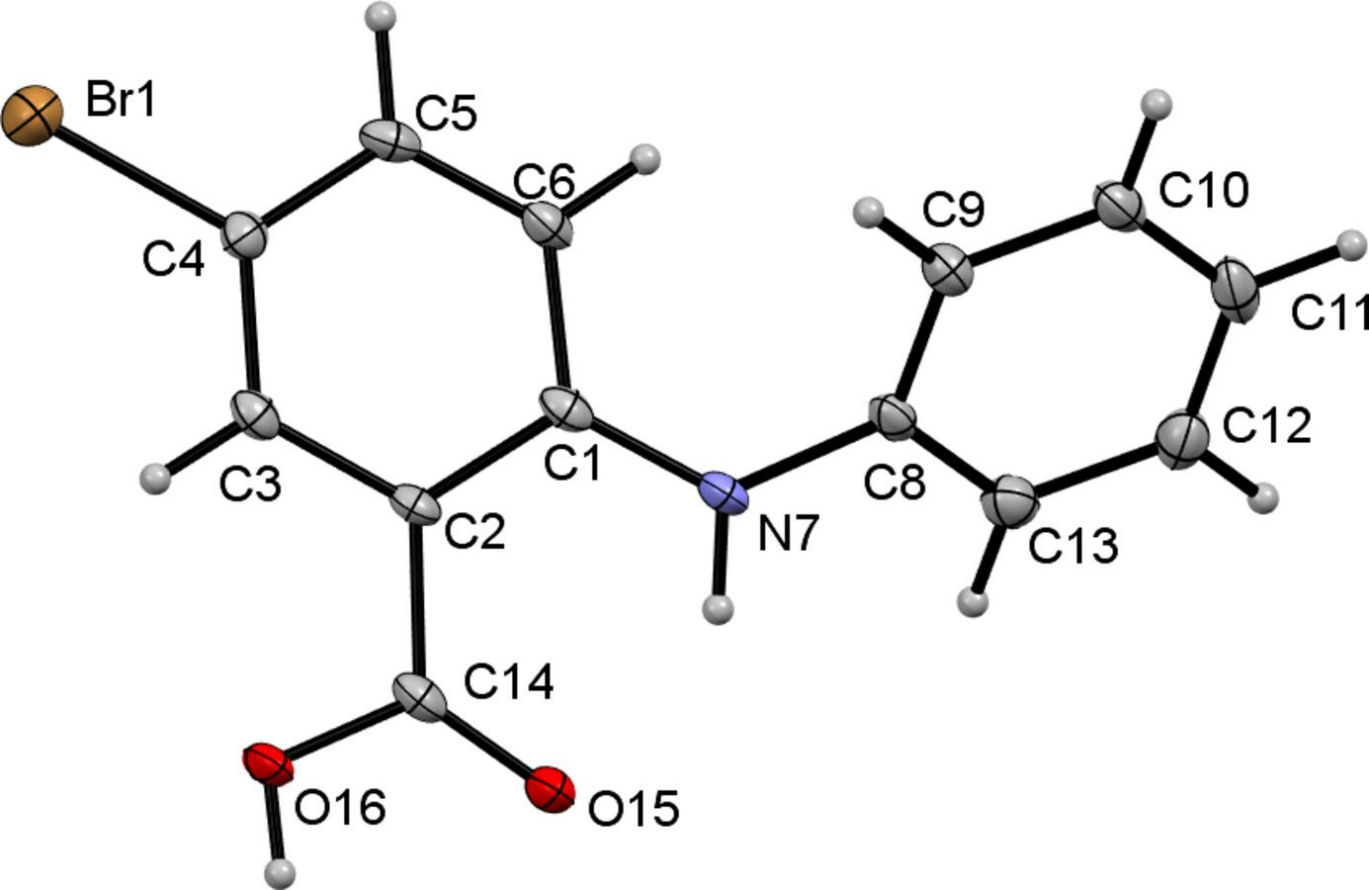
The mol­ecular structure of the title compound with displacement ellipsoids drawn at the 50% probability level.

**Figure 2:**
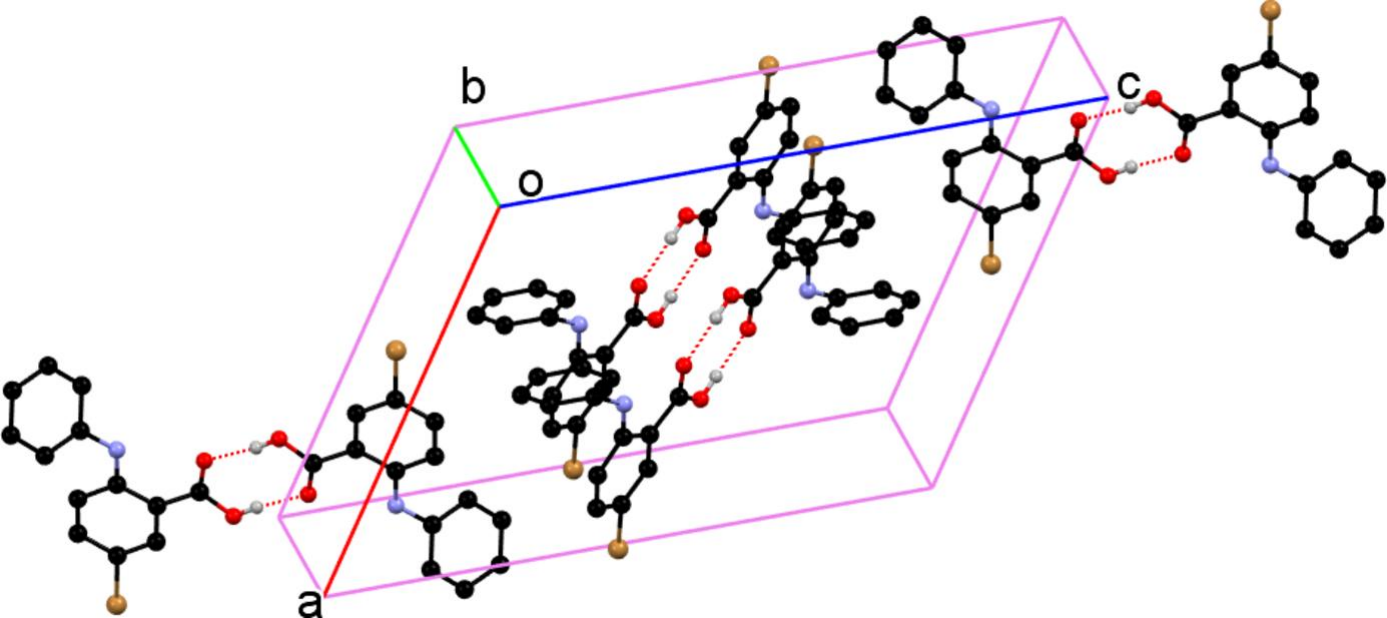
Packing of the mol­ecules in the title compound (for clarity, H atoms not involved in inter­molecular hydrogen bonding are omitted).

As part of our studies in this area, we now describe the synthesis by the Ullman reaction (Wolf *et al.*, 2006 ▸) and the crystal structure of the title compound, C_13_H_10_BrNO_2_. As a result of steric repulsion, the C1–C6 and C8–C13 aromatic rings are twisted, subtending a dihedral angle of 45.39 (11)°. An intra­molecular N7—H7⋯O15 hydrogen bond is seen (Fig. 1 ▸, Table 1 ▸). In the extended structure, the mol­ecules pair up to form carb­oxy­lic acid inversion dimers linked by pairs of O16—H16⋯O15 hydrogen bonds (Fig. 2 ▸, Table 1 ▸).

## Synthesis and crystallization

**Figure 3:**
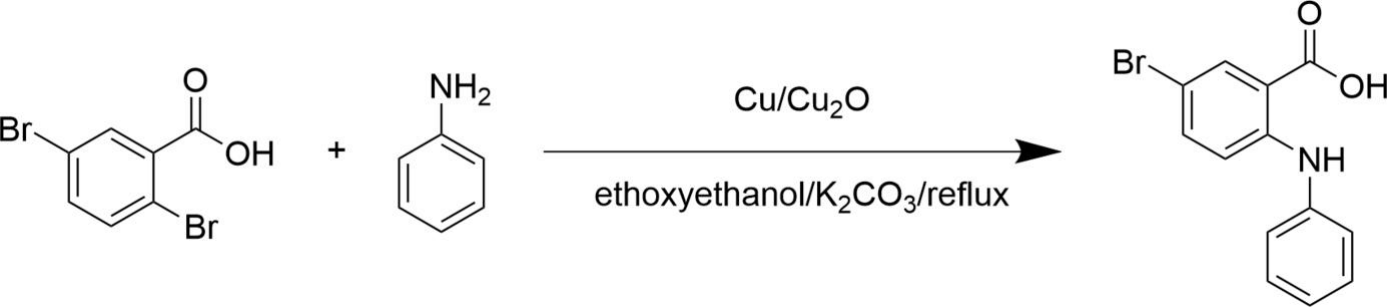
Synthesis of the title compound.

The title compound was prepared by reacting 2,5-di­bromo­benzoic acid and aniline in the presence of a catalyst at 403 K (Fig. 3 ▸). The product was purified by column chromatography. Single crystals were obtained by slowly evaporating an acetone solution of the title compound.

## Refinement

Crystal data, data collection and structure refinement details are summarized in Table 2 ▸.

## Supplementary Material

Crystal structure: contains datablock(s) global, I. DOI: 10.1107/S2414314624001986/hb4456sup1.cif

Structure factors: contains datablock(s) I. DOI: 10.1107/S2414314624001986/hb4456Isup2.hkl

Supporting information file. DOI: 10.1107/S2414314624001986/hb4456Isup3.cml

CCDC reference: 2336150

Additional supporting information:  crystallographic information; 3D view; checkCIF report

## Figures and Tables

**Table 1:** Hydrogen-bond geometry (Å, °)

*D*—H⋯*A*	*D*—H	H⋯*A*	*D*⋯*A*	*D*—H⋯*A*
N7—H7⋯O15	0.88	2.00	2.682 (4)	134
O16—H16⋯O15^i^	0.84	1.79	2.629 (4)	174

Symmetry code: (i) 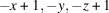 .

**Table 2:** Experimental details

Crystal data	
Chemical formula	C_13_H_10_BrNO_2_
*M*_r_	292.13
Crystal system, space group	Monoclinic, *P*2_1_/*n*
Temperature (K)	90
*a*, *b*, *c* (Å)	15.2054 (3), 3.8818 (1), 19.8109 (4)
β (°)	107.0391 (10)
*V* (Å^3^)	1118.00 (4)
*Z*	4
Radiation type	Mo *K*α
μ (mm^−1^)	3.66
Crystal size (mm)	0.20 × 0.10 × 0.05

Data collection	
Diffractometer	Nonius KappaCCD diffractometer
Absorption correction	Multi-scan (*SCALEPACK*; Otwinowski & Minor, 1997 ▸)
*T*_min_, *T*_max_	0.528, 0.838
No. of measured, independent and observed [*I* > 2σ(*I*)] reflections	4587, 2550, 2206
*R*_int_	0.026
(sin θ/λ)_max_ (Å^−1^)	0.649

Refinement	
*R*[*F* ^2^ > 2σ(*F* ^2^)], *wR*(*F* ^2^), *S*	0.038, 0.130, 1.21
No. of reflections	2550
No. of parameters	155
H-atom treatment	H-atom parameters constrained
Δρ_max_, Δρ_min_ (e Å^−3^)	0.67, −0.81

Computer programs: *COLLECT* (Nonius, 2002 ▸), *DENZO-SMN* (Otwinowski & Minor, 1997 ▸), *SHELXS97* and *SHELXL97* (Sheldrick, 2015 ▸) and *XP* in *SHELXTL* (Sheldrick, 2008 ▸).

## full crystallographic data

### Crystal data

**Table:**

C_13_H_10_BrNO_2_	*F*(000) = 584
*M**_r_* = 292.13	*D*_x_ = 1.736 Mg m^−^^3^
Monoclinic, *P*2_1_/*n*	Mo *K*α radiation, λ = 0.71073 Å
Hall symbol: -P 2yn	Cell parameters from 2931 reflections
*a* = 15.2054 (3) Å	θ = 1.0–27.5°
*b* = 3.8818 (1) Å	µ = 3.66 mm^−^^1^
*c* = 19.8109 (4) Å	*T* = 90 K
β = 107.0391 (10)°	Rod, yellow
*V* = 1118.00 (4) Å^3^	0.20 × 0.10 × 0.05 mm
*Z* = 4	

### Data collection

**Table:**

Nonius KappaCCD diffractometer	2550 independent reflections
Radiation source: fine-focus sealed tube	2206 reflections with *I* > 2σ(*I*)
Graphite monochromator	*R*_int_ = 0.026
Detector resolution: 18 pixels mm^-1^	θ_max_ = 27.5°, θ_min_ = 1.5°
ω scans at fixed χ=55°	*h* = −19→19
Absorption correction: multi-scan (Scalepack; Otwinowski & Minor, 1997)	*k* = −4→5
*T*_min_ = 0.528, *T*_max_ = 0.838	*l* = −25→25
4587 measured reflections	

### Refinement

**Table:**

Refinement on *F*^2^	Primary atom site location: structure-invariant direct methods
Least-squares matrix: full	Secondary atom site location: difference Fourier map
*R*[*F*^2^ > 2σ(*F*^2^)] = 0.038	Hydrogen site location: inferred from neighbouring sites
*wR*(*F*^2^) = 0.130	H-atom parameters constrained
*S* = 1.21	*w* = 1/[σ^2^(*F*_o_^2^) + (0.0621*P*)^2^ + 2.3209*P*] where *P* = (*F*_o_^2^ + 2*F*_c_^2^)/3
2550 reflections	(Δ/σ)_max_ < 0.001
155 parameters	Δρ_max_ = 0.67 e Å^−^^3^
0 restraints	Δρ_min_ = −0.81 e Å^−^^3^

### Special details

**Table:**

Geometry. All esds (except the esd in the dihedral angle between two l.s. planes) are estimated using the full covariance matrix. The cell esds are taken into account individually in the estimation of esds in distances, angles and torsion angles; correlations between esds in cell parameters are only used when they are defined by crystal symmetry. An approximate (isotropic) treatment of cell esds is used for estimating esds involving l.s. planes.
Refinement. Refinement of F^2^ against ALL reflections. The weighted R-factor wR and goodness of fit S are based on F^2^, conventional R-factors R are based on F, with F set to zero for negative F^2^. The threshold expression of F^2^ > 2sigma(F^2^) is used only for calculating R-factors(gt) etc. and is not relevant to the choice of reflections for refinement. R-factors based on F^2^ are statistically about twice as large as those based on F, and R- factors based on ALL data will be even larger. The positions of the H atoms attached to N1 and O1 were obtained from a difference Fourier map. The other H atoms were positioned geometrically with C—H = 0.95 Å and constrained to ride on their parent atoms with *U*_iso_(H) = 1.2*U*_eq_(C,*N*) or 1.5*U*_eq_(O).

### Fractional atomic coordinates and isotropic or equivalent isotropic displacement parameters (Å^2^)

**Table:**

	*x*	*y*	*z*	*U*_iso_*/*U*_eq_	
Br1	0.10141 (2)	0.46781 (11)	0.57989 (2)	0.02709 (16)	
C1	0.4236 (2)	0.5204 (8)	0.66869 (17)	0.0142 (7)	
C2	0.3806 (2)	0.3697 (9)	0.60154 (16)	0.0129 (6)	
C3	0.2839 (2)	0.3583 (9)	0.57554 (16)	0.0142 (6)	
H3	0.2554	0.2540	0.5311	0.017*	
C4	0.2307 (2)	0.4972 (9)	0.61400 (17)	0.0149 (7)	
C5	0.2722 (2)	0.6592 (9)	0.67881 (17)	0.0154 (7)	
H5	0.2351	0.7620	0.7044	0.018*	
C6	0.3662 (2)	0.6696 (9)	0.70531 (16)	0.0155 (7)	
H6	0.3933	0.7796	0.7493	0.019*	
N7	0.5173 (2)	0.5306 (8)	0.69514 (15)	0.0172 (6)	
H7	0.5482	0.4840	0.6650	0.021*	
C8	0.5710 (2)	0.6066 (9)	0.76502 (17)	0.0149 (7)	
C9	0.5449 (2)	0.4996 (9)	0.82367 (18)	0.0162 (7)	
H9	0.4883	0.3826	0.8176	0.019*	
C10	0.6029 (3)	0.5664 (9)	0.89132 (18)	0.0192 (7)	
H10	0.5851	0.4970	0.9314	0.023*	
C11	0.6858 (3)	0.7323 (10)	0.90048 (18)	0.0221 (8)	
H11	0.7246	0.7782	0.9467	0.026*	
C12	0.7125 (2)	0.8319 (10)	0.84229 (19)	0.0214 (7)	
H12	0.7704	0.9403	0.8487	0.026*	
C13	0.6547 (2)	0.7736 (10)	0.77441 (18)	0.0195 (7)	
H13	0.6723	0.8478	0.7346	0.023*	
C14	0.4345 (2)	0.2211 (9)	0.55789 (16)	0.0156 (7)	
O15	0.51870 (16)	0.2210 (7)	0.57446 (12)	0.0211 (6)	
O16	0.38326 (17)	0.0837 (7)	0.49767 (12)	0.0199 (5)	
H16	0.4176	−0.0037	0.4759	0.030*	

### Atomic displacement parameters (Å^2^)

**Table:**

	*U*^11^	*U*^22^	*U*^33^	*U*^12^	*U*^13^	*U*^23^
Br1	0.0193 (2)	0.0366 (3)	0.0254 (2)	0.00184 (16)	0.00662 (16)	−0.00424 (16)
C1	0.0205 (16)	0.0130 (16)	0.0102 (14)	−0.0001 (13)	0.0061 (12)	0.0015 (12)
C2	0.0175 (15)	0.0140 (15)	0.0089 (13)	−0.0007 (13)	0.0063 (12)	0.0002 (12)
C3	0.0191 (16)	0.0128 (15)	0.0104 (14)	0.0001 (13)	0.0036 (12)	0.0012 (12)
C4	0.0149 (15)	0.0170 (17)	0.0130 (15)	0.0015 (13)	0.0043 (12)	0.0030 (12)
C5	0.0232 (17)	0.0128 (16)	0.0132 (14)	0.0021 (14)	0.0100 (13)	0.0008 (13)
C6	0.0222 (17)	0.0144 (16)	0.0097 (13)	0.0028 (14)	0.0041 (12)	−0.0003 (12)
N7	0.0181 (14)	0.0250 (17)	0.0095 (12)	−0.0002 (12)	0.0054 (11)	−0.0044 (11)
C8	0.0179 (16)	0.0148 (16)	0.0114 (14)	0.0014 (13)	0.0034 (12)	−0.0037 (13)
C9	0.0180 (16)	0.0171 (17)	0.0130 (15)	−0.0004 (13)	0.0039 (12)	−0.0013 (13)
C10	0.0238 (18)	0.0208 (18)	0.0122 (15)	0.0067 (14)	0.0040 (13)	−0.0006 (13)
C11	0.0246 (18)	0.0205 (19)	0.0153 (15)	0.0074 (15)	−0.0033 (13)	−0.0035 (14)
C12	0.0169 (16)	0.0216 (19)	0.0235 (17)	0.0012 (15)	0.0023 (14)	−0.0037 (15)
C13	0.0209 (17)	0.0201 (18)	0.0180 (16)	0.0027 (14)	0.0066 (13)	−0.0011 (14)
C14	0.0219 (17)	0.0159 (16)	0.0094 (13)	−0.0006 (14)	0.0053 (12)	−0.0002 (12)
O15	0.0161 (12)	0.0342 (15)	0.0135 (11)	−0.0013 (11)	0.0052 (9)	−0.0074 (11)
O16	0.0169 (12)	0.0315 (15)	0.0123 (11)	−0.0035 (11)	0.0058 (9)	−0.0096 (10)

### Geometric parameters (Å, º)

**Table:**

Br1—C4	1.885 (3)	C8—C13	1.390 (5)
C1—N7	1.368 (5)	C8—C9	1.397 (5)
C1—C6	1.412 (5)	C9—C10	1.396 (5)
C1—C2	1.424 (4)	C9—H9	0.9500
C2—C3	1.409 (5)	C10—C11	1.379 (6)
C2—C14	1.471 (4)	C10—H10	0.9500
C3—C4	1.374 (5)	C11—C12	1.385 (5)
C3—H3	0.9500	C11—H11	0.9500
C4—C5	1.403 (5)	C12—C13	1.393 (5)
C5—C6	1.371 (5)	C12—H12	0.9500
C5—H5	0.9500	C13—H13	0.9500
C6—H6	0.9500	C14—O15	1.226 (4)
N7—C8	1.417 (4)	C14—O16	1.330 (4)
N7—H7	0.8800	O16—H16	0.8400
			
N7—C1—C6	121.5 (3)	C13—C8—N7	118.1 (3)
N7—C1—C2	120.7 (3)	C9—C8—N7	121.9 (3)
C6—C1—C2	117.7 (3)	C10—C9—C8	119.3 (3)
C3—C2—C1	119.8 (3)	C10—C9—H9	120.3
C3—C2—C14	118.3 (3)	C8—C9—H9	120.3
C1—C2—C14	121.9 (3)	C11—C10—C9	120.6 (3)
C4—C3—C2	120.4 (3)	C11—C10—H10	119.7
C4—C3—H3	119.8	C9—C10—H10	119.7
C2—C3—H3	119.8	C10—C11—C12	120.0 (3)
C3—C4—C5	120.2 (3)	C10—C11—H11	120.0
C3—C4—Br1	120.0 (3)	C12—C11—H11	120.0
C5—C4—Br1	119.9 (3)	C11—C12—C13	120.1 (4)
C6—C5—C4	120.2 (3)	C11—C12—H12	119.9
C6—C5—H5	119.9	C13—C12—H12	119.9
C4—C5—H5	119.9	C8—C13—C12	119.9 (3)
C5—C6—C1	121.5 (3)	C8—C13—H13	120.0
C5—C6—H6	119.2	C12—C13—H13	120.0
C1—C6—H6	119.2	O15—C14—O16	122.1 (3)
C1—N7—C8	128.2 (3)	O15—C14—C2	124.1 (3)
C1—N7—H7	115.9	O16—C14—C2	113.8 (3)
C8—N7—H7	115.9	C14—O16—H16	109.5
C13—C8—C9	119.9 (3)		
			
N7—C1—C2—C3	179.3 (3)	C1—N7—C8—C13	−147.3 (4)
C6—C1—C2—C3	−3.1 (5)	C1—N7—C8—C9	36.8 (5)
N7—C1—C2—C14	−0.3 (5)	C13—C8—C9—C10	0.9 (5)
C6—C1—C2—C14	177.3 (3)	N7—C8—C9—C10	176.7 (3)
C1—C2—C3—C4	1.1 (5)	C8—C9—C10—C11	−0.9 (5)
C14—C2—C3—C4	−179.3 (3)	C9—C10—C11—C12	−0.5 (6)
C2—C3—C4—C5	1.6 (5)	C10—C11—C12—C13	1.8 (6)
C2—C3—C4—Br1	−178.1 (3)	C9—C8—C13—C12	0.4 (5)
C3—C4—C5—C6	−2.2 (5)	N7—C8—C13—C12	−175.6 (3)
Br1—C4—C5—C6	177.4 (3)	C11—C12—C13—C8	−1.7 (6)
C4—C5—C6—C1	0.2 (5)	C3—C2—C14—O15	178.8 (3)
N7—C1—C6—C5	−180.0 (3)	C1—C2—C14—O15	−1.6 (6)
C2—C1—C6—C5	2.4 (5)	C3—C2—C14—O16	−1.2 (5)
C6—C1—N7—C8	15.9 (5)	C1—C2—C14—O16	178.4 (3)
C2—C1—N7—C8	−166.6 (3)		

### Hydrogen-bond geometry (Å, º)

**Table:**

*D*—H···*A*	*D*—H	H···*A*	*D*···*A*	*D*—H···*A*
N7—H7···O15	0.88	2.00	2.682 (4)	134
O16—H16···O15^i^	0.84	1.79	2.629 (4)	174

Symmetry code: (i) −*x*+1, −*y*, −*z*+1.
